# Physical conditioning and mental stress reduction - a randomised trial in patients undergoing cardiac surgery

**DOI:** 10.1186/1472-6882-11-20

**Published:** 2011-03-09

**Authors:** Franklin Rosenfeldt, Lesley Braun, Ondine Spitzer, Scott Bradley, Judy Shepherd, Michael Bailey, Juliana van der Merwe, Jee -Yoong Leong, Donald Esmore

**Affiliations:** 1 Cardiac Surgical Research Unit, Alfred Hospital, Department of Surgery, Monash University, Baker IDI Institute Melbourne, Australia; 2 Department of Epidemiology & Preventive Medicine, Monash University, Melbourne, Australia; 3 Department of Physiotherapy, Alfred Hospital, Melbourne, Australia; 4 Occupational Therapy Services, Alfred Hospital, Melbourne, Australia

## Abstract

**Background:**

Preoperative anxiety and physical unfitness have been shown to have adverse effects on recovery from cardiac surgery. This study involving cardiac surgery patients was primarily aimed at assessing the feasibility of delivering physical conditioning and stress reduction programs within the public hospital setting. Secondary aims were to evaluate the effect of these programs on quality of life (QOL), rates of postoperative atrial fibrillation (AF) and length of stay (LOS) in hospital.

**Methods:**

Elective patients scheduled for coronary artery bypass graft and/or valve surgery at a public hospital in Melbourne, Australia were enrolled. Patients were randomized to receive either holistic therapy (HT) or usual care (UC). HT consisted of a series of light physical exercise sessions together with a mental stress reduction program administered in an outpatient setting for the first two weeks after placement on the waiting list for surgery. A self-administered SF-36 questionnaire was used to measure QOL and hospital records to collect data on LOS and rate of postoperative AF.

**Results:**

The study population comprised 117 patients of whom 60 received HT and 57 received UC. Both programs were able to be delivered within the hospital setting but ongoing therapy beyond the two week duration of the program was not carried out due to long waiting periods and insufficient resources. HT, as delivered in this study, compared to UC did not result in significant changes in QOL, LOS or AF incidence.

**Conclusions:**

Preoperative holistic therapy can be delivered in the hospital setting, although two weeks is insufficient to provide benefits beyond usual care on QOL, LOS or postoperative AF. Further research is now required to determine whether a similar program of longer duration, or targeted to high risk patients can provide measurable benefits.

**Trial registration:**

This trial was conducted as part of a larger study and according to the principles contained in the CONSORT statement 2001.

## Background

Undergoing coronary artery bypass graft surgery (CABG) is a significant life event with an important psycho-emotional impact on patients and their families [1]. Most patients report fear and anxiety [2] and many report that uncertainty about the future is more disturbing than their chest pain [3]. Functional and psychological status has been shown to deteriorate while on the waiting list for cardiac surgery [4]. The longer a patient stays on the waiting list for cardiac surgery, the more likely they are to reduce their leisure activities, causing them to experience anxiety, reduced physical and social functioning, poorer vitality, and general health [5-7]. Preoperative anxiety is a predictor of poor recovery from cardiac surgery [7]. Depression is a common problem after cardiac surgery [8].

A number of studies have demonstrated positive influence of mindfulness-based stress reduction techniques on physiological processes and psychological states, such as reducing blood pressure, increasing pain tolerance and improving coping ability [9,10]. A review of preoperative relaxation interventions in surgical patients supports the use of simple techniques such as deep breathing [11] and self-hypnosis [12] to enhance a range of postoperative outcomes. Mind-body approaches such as lifestyle modification and stress reduction have long been known to have beneficial effects on cardiovascular diseases [13]. The Columbia-Presbyterian Medical Center in the USA utilizes a range of stress reduction techniques for surgical patients [14], including music therapy, hypnotherapy, yoga and massage in the cardiac surgery department and report positive benefits. In particular their trials have demonstrated significant reduction in anxiety and postoperative pain using hypnosis in the perioperative cardiac surgery setting [15]. We are not aware of any other intervention studies specifically investigating the usefulness of mental stress reduction practices in cardiac surgery patients.

Findings from a previous study using an exercise program to improve fitness of CABG patients support the commonsense notion that preoperative physical conditioning of cardiac patients would lead to quicker recovery postoperatively [4]. Other studies have demonstrated positive cardiovascular effects of preoperative exercise on surgical patients [16] and psychological benefits of postoperative exercise on CABG patients [17].

Previously we conducted a pilot study to assess the feasibility of utilising a combination of preoperative physical conditioning and mental stress reduction combined with metabolic therapy in cardiac surgery patients [18]. The aim of the present study was to confirm the feasibility of delivering these programs to a larger number of patients in the public hospital setting. Secondary aims were to evaluate the effects of preoperative physical conditioning and stress reduction on postoperative quality of life (QOL), incidence of postoperative atrial fibrillation (AF) and length of hospital stay (LOS).

## Methods

Patients undergoing elective coronary artery bypass graft (CABG) and/or valve surgery at the Alfred Hospital, a major public hospital in Melbourne Australia, between November 2004 and June 2006 were enrolled into the study. A total of 137 patients were assessed for possible enrolment. Of these a total of 20 were excluded, including 14 who failed to meet all inclusion criteria, four who refused for a variety of reasons, two patients who did not receive the allocated treatment, thus leaving 117 patients in the study. None were lost to follow-up. Patients were randomised, using a computer-generated code, to receive either usual care (UC) or holistic therapy (HT). Those selected for HT participated in a preoperative series of light physical exercise sessions together with a mental stress reduction program. Patients allocated to UC were used as a control group and awaited surgery at home without receiving additional therapy. Patients were excluded if they had urgent or emergency surgery, severe aortic valve stenosis (a mean gradient of greater than 35 mmHg, and thus be at risk during exercise), limited English (which would hinder the administration of the Short Form 36-item (SF-36) Health Survey Questionnaire), or were in NYHA class IV heart failure.

The SF-36 questionnaire consists of 36 questions which measure eight domains: physical functioning; role limitations owing to physical problems; role limitations owing to emotional problems; social functioning; mental health; general health perceptions; vitality; and bodily pain [19]. In our previous work in using the SF-36 questionnaire we found that the physical and mental parameters of the composite scores, rather than individual domains, were most useful as methods to describe QOL [20].

The primary endpoint was a change in mental and physical quality of life, measured by the SF-36. Secondary endpoints were length of hospital stay (LOS) and rate of postoperative atrial fibrillation (AF), as determined from hospital records.

The study protocol was approved by the Alfred Hospital Ethics Committee and informed consent was obtained from patients before participation. The trial was conducted according to the principles contained in the CONSORT statement 2001 [21].

### Physical exercise program

The physical exercise program was based on the American College of Sports Medicine guidelines [22]which recognise that any person with an initially low level of fitness, as is often the case in those awaiting cardiac surgery, may achieve fitness improvements with relatively low intensity aerobic exercise training (approximately 40 to 50% of maximum oxygen uptake reserve or heart rate reserve). Our physical exercise program was conducted as outpatient sessions for the first two weeks on the waiting list and was supervised by a physiotherapist. Patients participated in two 60-minute exercise sessions per week. The first session consisted of gentle stretching, 15 minutes of cycling on a stationary bicycle followed by 10-15 minutes of walking on a treadmill. Patients exercised until they reached a maximum of 60% of their expected maximum heart rate (HR_max _= 220 - age). To ensure patient safety, a physician was present at each patient's first exercise session utilising ECG and heart rate monitoring. During each subsequent session participants undertook 40 minutes of an endurance-type exercise circuit that included three exercises: cycle ergometry, treadmill walking and arm ergometry, with heart rate closely monitored by the physiotherapist.

In addition to the supervised sessions, patients were encouraged to complete at least 30 minutes (to a maximum of one hour) of continuous aerobic exercise on at least two other occasions during the week. Patients used heart rate monitors to judge the level of activity required to attain their target heart rate.

After the two week program was completed, patients were encouraged to continue regular physical exercise (mainly in the form of walking) at home for at least 30 minutes on four days per week until surgery. Participants were provided with a heart rate monitor to ensure they were reaching the desired level of exercise intensity.

### Mental stress reduction

An occupational therapist with extensive experience in stress reduction techniques in cardiac surgery patients conducted mental stress reduction therapy [3,23] as outpatient sessions for patients' first two weeks on the waiting list. Four individualised 60-minute sessions were provided to each patient and family members were encouraged to attend. Each session consisted of education about the effects and management of stress and an experiential component involving relaxation techniques such as deep breathing exercises and meditation. Participants were taught to recognise the situations that caused them stress and practical ways to either accept or avoid these situations. Participants were given homework and handouts after each session and were encouraged to practise different relaxation techniques daily at home until the time of their surgery. To augment this program, patients were also provided with a CD of relaxing music and were encouraged to listen to this for 20 minutes each day as part of their relaxation practice.

### Outcome measurements

Quality of life measures were obtained via the Short Form 36-item Health Survey Questionnaire (SF-36) which was self-administered at three different time points: commencement of the program (baseline), immediately preoperatively and 6 weeks after surgery. Completed questionnaires were then scored on the online calculator http://www.sf-36.org/demos/SF-36v2.html to generate physical and mental summary scores. Hospital length of stay and rate of postoperative AF were determined using the patients' medical records.

### Statistical analysis

The required sample size was calculated from the results of our previous pilot study [18]. It was calculated that to detect a change of 20% in physical quality of life score this study would require 60 subjects per group to have an 80% power with a two-sided p-value of 0.05. These calculations were based on an observed difference of 10 units and a standard deviation of 19.6.

Parametric continuous variables are expressed as mean ± standard error and non-parametric continuous variables are expressed as median (interquartile range). Comparisons of differences in proportions were performed using chi-square (χ^2^) or Fischer's exact tests, and univariate comparisons of continuous variables were performed using the Student's *t*-test for parametric data and Wilcoxon rank-sum test for non-parametric data. Variables with repeated measures were analysed using the two-way repeated measures analysis of variance (ANOVA) fitting a group and time effect and a group by time interaction to ascertain if the two groups responded differently over time.

## Results

A comparison of demographics between the Holistic Therapy group and the Usual Care group showed that there were no significant differences in any of the baseline parameters (Table 1).

**Table 1:** Patient demographics

	Holistic therapy (*n *= 60)	Usual care (*n *= 57)	*p *Value
Age (median years)	62.5 (59.0-68.5)	68 (58.0-77.0)	0.06
Male gender (%)	78%	70%	0.31
Diabetes mellitus	20%	29%	0.22
Previous MI	28%	31%	0.70
EuroSCORE	2.5 (1.5-6.1)	3.1 (1.6-7.6)	0.23
ASA Classification	3 (2-3)	3 (3-3)	0.32
CABG alone (n = 71)	37 (62%)	34 ((60%)	0.82
Valve (± CABG)	23 (38%)	23 (40%)	0.82

MI = myocardial infarction; EuroSCORE = European System for Cardiac Operative Risk Evaluation; ASA = American Society of Anaesthesiologists (Physical Status Classification System); CABG = coronary artery bypass graft

We found that delivering a holistic program which incorporated physical conditioning and mental stress reduction to cardiac surgery patients was possible within a public hospital. Patients were able to tolerate the physical exercise program and participate in the mental stress reduction program despite having severe cardiac disease requiring surgery.

There were no significant between-group differences in the clinical endpoints of rate of atrial fibrillation or hospital length of stay (Table 2).

**Table 2:** Clinical endpoints

	Holistic therapy (*n *= 60)	Usual care (*n *= 57)	*p *Value
Troponin at 24 hr (μg/L)	1.64 (0.75-4.15)	1.47 (1-2.79)	0.91

Atrial fibrillation (%)	36%	33%	0.71

Length of stay (days)	6 (5-8)	6 (5-8)	0.54

Physical quality of life in both groups was significantly improved 6 weeks after surgery compared to pre-treatment baseline (p < 0.001) but there was no significant difference between groups overall (p = 0.35) and no significant difference between the groups over time (p = 0.99) (Table 3).

**Table 3:** Quality of life measurements at different time points

	Holistic therapy (*n *= 60)	Usual care (*n *= 57)	*p *Value
**SF-36 Physical composite score**			
Baseline	40.4 ± 1.2	38.9 ± 1.2	0.40
Preoperative (post-therapy)	40.6 ± 1.1	39.4 ± 1.3	0.49
Postoperative (6 weeks)	44.1 ± 1.0*	42.8 ± 1.3*	0.45
**SF-36 Mental composite score**			
Baseline	43.3 ± 1.6	44.3 ± 1.3	0.62
Preoperative (post-therapy)	45.6 ± 1.4	43.1 ± 1.4	0.22
Postoperative (6 weeks)	45.4 ± 1.2**	45.2 ± 1.6	0.91

Parametric data presented as mean ± standard error of the mean (SEM).

* significant difference compared to baseline (p < 0.001)

** significant difference compared to baseline (p = 0.03)

Mental quality of life 6 weeks after surgery compared to pre-treatment baseline was significantly improved in the holistic group (p = 0.03) but remained unchanged in the usual care group (p = 0.84). There was, however, no difference between groups overall (p = 0.46) and no significant difference between groups over time (p = 0.16) (Table 2).

## Discussion

Elective cardiac surgery patients attending the Alfred Hospital received either preoperative physical and mental stress preparation or usual care in an effort to assess whether this holistic approach could be delivered in a public hospital setting and whether it would favourably alter recovery after cardiac surgery. The study demonstrated that it is feasible to deliver physical conditioning and mental stress reduction programs at a public hospital and that patients with advanced cardiac disease are able to participate safely. However, long waiting lists and a shortage of resources available to implement the program for longer than two weeks presented logistic difficulties. No significant differences were found between the group receiving usual care or holistic therapy for QOL, LOS or post-surgery AF rates. However there was an indication of improved mental QOL with holistic therapy but not with usual care.

Patients presenting for cardiac surgery in the current era are more elderly and have more co-morbidities than in the past [24]. As a result they tend to be more unfit, have more postoperative complications and require a longer convalescence period than patients in the past [25]. Importantly, these older, sicker patients are disproportionately represented in morbidity and mortality statistics [26].

Surgery is a major stressor for these patients with decreased functional reserve causing loss of muscle mass, deconditioning, hypoxaemia, mental disturbances and sleep disorders. Anxiety, depression and stress are associated with greater morbidity after cardiac surgery [8,27]. Further, the often lengthy period waiting for cardiac surgery can exacerbate stress and anxiety [28], and adversely affect physical and social functioning [6]. Thus current cardiac surgery patients might benefit from preoperative interventions to promote recovery and reduce surgical complications. The preoperative waiting period provides a window of opportunity to support CABG patients and influence postoperative outcomes [4]. Indeed CABG patients have reported positive experiences with preoperative support programs, which may include education, encouragement to achieve behavioural changes, regular exercise and relaxation training [29]. The preoperative period as well as the in-hospital stay can provide an opportunity to encourage patients to attend postoperative rehabilitation with consequent improvement in recovery time and return to work. During the current study the participation rate in postoperative rehabilitation was 59%. We have now increased this rate to 86% by incorporating information about, and availability of postoperative rehabilitation programs in our new Cardiac Wellness Program.

A previous pilot study conducted by our unit in 16 cardiac surgery patients suggested that a preoperative holistic program of physical conditioning and mental stress reduction, combined with antioxidant supplementation, can result in significant improvements one month after surgery in physical and mental QOL scores as measured by the SF-36 questionnaire [18]. The original holistic program consisted of a home-based physical exercise program and hospital-based mental stress reduction sessions. To encourage participation and monitor compliance, patients were contacted by telephone twice a week and visited at home at least once between study enrolment and day of surgery.

The current and previous holistic programs are very similar in many aspects however they differed in two important ways. Firstly, patients in the current study did not receive twice weekly phone calls or home visits to provide encouragement and monitor compliance. The lack of support during the lengthy waiting period may have reduced patient compliance, however the lack of compliance monitoring means we are unable to confirm this suspicion. Poor patient compliance during the waiting period could be reasonably expected to influence study results as any small benefits achieved in the two weeks of therapy would be dissipated over the two months on average between enrolment into the program and surgery.

Secondly, home-based exercise programs may be more acceptable to patients and provide a seamless method of ensuring compliance during the waiting period when access to hospital facilities is no longer available. As such, it is suggested that future studies incorporate ongoing patient support by providing regular patient contact during the waiting period and physical exercise programs be delivered in the home or within a cardiac rehabilitation centre near to their home where patients can continue them with little assistance.

### Physical exercise

It is well established that regular physical exercise in the elderly, including heart disease patients, improves cardiorespiratory and other physiological functions, with an accompanying improvement in physical performance [30-32]. For instance, patients with chronic heart failure who perform regular moderate exercise have increased functional capacity, greater muscle strength, lower mortality and greater quality of life compared to non-exercising controls [33-35]. Exercise training has also been found to significantly reduce anxiety [36].

Preoperative fitness is a predictor of postoperative outcomes and poor fitness scores are associated with more complications and longer hospital stays [25]. However there is little research on preoperative physical exercise conditioning programs in surgical patients [28], an approach described as 'prehabilitation' [37]. The few randomised studies investigating the effects of physical exercise training on surgical patients in the preoperative setting suggest that prehabilitation can be effective in improving surgical outcome [16].

Exercise prehabilitation prior to abdominal or cardiac surgery has been shown to reduce postoperative complications, reduce length of hospital stay, improve quality of life, and reduce the decline in functional disability compared to sedentary controls [16,28]. An important study of CABG patients in the mid-1990 s tested preoperative exercise training in patients having CABG surgery [4]. Treated patients received individualised and supervised exercise training twice weekly for a minimum of 8 weeks, alongside education, monthly telephone calls and encouragement to participate postoperatively in an existing cardiac rehabilitation program. The study demonstrated positive benefit including a one day reduction in length of total postoperative hospital stay and an improvement in physical quality of life 6-8 weeks postoperatively compared to placebo, with advantage still present 6 months postoperatively. However, anxiety levels were no lower in the treated group through the waiting period. This study showed that preoperative exercise training in patients awaiting CABG surgery was not only safe and viable, but its implementation accelerated recovery and conferred quality of life benefits to these patients.

The amount of time it takes to improve physical conditioning depends on the intensity, duration and frequency of exercise, but it usually takes at least 6 weeks to see positive results even under optimal conditions. As the average waiting period for surgery for our study participants was 70 days, this would have provided ample time to have a positive impact on physical conditioning if an exercise program had been maintained regularly. However there was neither facility to provide ongoing coaching by our holistic team after the initial two weeks of therapy nor any assessment of compliance. Our previous pilot study included twice weekly telephone coaching and home visits by a member of the research team, providing motivation and encouragement. This was not possible in the current study due to funding limitations. A longer period of a supervised exercise program, perhaps targeting patients with preoperative physical limitations, would be more likely to have a positive impact in cardiac surgery.

### Mental stress reduction

Preoperative anxiety symptoms are significantly associated with increased surgical mortality [27]. Depression is associated with increased cardiac morbidity and mortality in CABG patients [27]. Further, previous studies have shown that long preoperative waiting periods have adverse psychological effects on CABG patients [38] and hospitalisation itself increases stress and anxiety and that this that may impact on recovery from heart surgery [38-40]. Some studies have demonstrated that anxiety and depression may contribute to poorer pain relief after surgery [41,42]. Furthermore preoperative anxiety and postoperative depression increases postoperative readmission rates more than twofold [43]. Tully et. al found that postoperative but not preoperative anxiety was associated with an increased rate of postoperative AF [44].

As well as concerns about their health and impending cardiac surgery CABG patients have other factors to negotiate preoperatively, such as long waiting periods. It has been shown that in the waiting period patients experience high levels of stress, fear and anxiety [2,45,46]. Uncertainty about when surgery will occur is an added stress for patients awaiting CABG surgery. Our study did not demonstrate that intervention reduced the incidence of postoperative morbidities such as AF after cardiac surgery, perhaps due to the truncated stress reduction program we were able to offer.

Although the results of this present study were not significant we still believe that a program of preoperative stress reduction therapy in cardiac surgery patients might provide them with a coping strategy to reduce anxiety and depression, particularly in patients with indications of these problems before surgery. Improving these psycho-emotional factors may also reduce postoperative complications and length of stay and improve postoperative quality of life. From this feasibility study we have clear direction as to how to better design a program targeting both physical fitness and mental stress.

## Conclusions

There is scope for further trials utilizing physical exercise and mental stress reduction for cardiac surgery patients. It is reasonable to expect that improvements in QOL parameters would require longer than two weeks supervised preoperative intervention, "prehabilitation", combined with a longer than six week postoperative period for follow-up. Particular attention needs to be paid to overcoming the difficulties of implementing such a program in a public hospital setting and perhaps targeting older, high risk patients. Adequate resources utilising appropriate staff to provide ongoing support and encouragement to patients are essential to the success of a program of this nature. Evaluation of compliance is necessary to assess the validity of the program. We firmly believe that the concept of physical and mental prehabilitation is sound and warrants further investigation.

## Competing interests

The authors declare that they have no competing interests.

## Authors' contributions

FR designed the study, carried out the initial data analysis and drafted the manuscript. LB assisted with data analysis and assisted with writing the manuscript. OS assisted with data analysis and assisted with writing the manuscript. SB designed the exercise program and supervised its application in the physiotherapy department. JS designed the stress reduction program and administered it to all patients in the intervention group. MB carried out the statistical analysis. JVDM assisted with data collection and analysis. JYL assisted with design, implemented the study and assisted with data analysis and manuscript writing. DE assisted with the application of the program in the surgical unit. All authors have read and approved the final manuscript.

## Pre-publication history

The pre-publication history for this paper can be accessed here:

http://www.biomedcentral.com/1472-6882/11/20/prepub
